# Molecular-Level Insights into CO_2_ Dissolution Trapping in Deep Saline Aquifers: Diffusion Behavior in NaCl Brines

**DOI:** 10.3390/molecules31122043

**Published:** 2026-06-11

**Authors:** Tiankuo Zhou, Dexiang Li

**Affiliations:** 1 SDU-ANU Joint Science College, Shandong University, Weihai 264209, China; 202300700155@mail.sdu.edu.cn; 2 School of Nuclear Science, Energy and Power Engineering, Shandong University, Jinan 250061, China

**Keywords:** CO_2_ geological storage, saline aquifer, molecular dynamics simulation, diffusion coefficient, salt concentration

## Abstract

Carbon capture, utilization, and storage (CCUS) is critical for carbon neutrality, and deep saline aquifers are promising reservoirs for CO_2_ sequestration. CO_2_ diffusion in brine directly affects dissolution trapping efficiency and is strongly influenced by salt ions. Molecular dynamics simulations were employed to investigate CO_2_ diffusion in NaCl brines under varying concentrations (0.1–5.0 mol/L), temperatures (298–353 K), and pressures (3–40 MPa). Diffusion coefficients were derived from mean square displacement, and radial distribution functions combined with hydrogen bond analysis were used to elucidate microscopic mechanisms. Results show that as NaCl concentration increases from 0.1 to 5.0 mol/L, the diffusion coefficient decreases by ~50%, reflecting the kinetic consequence of the salting-out effect. Raising temperature from 298 to 353 K enhances diffusion by ~149%, following Arrhenius behavior, while pressure shows negligible influence below 30 MPa but causes a 15% drop at 40 MPa. RDF analysis reveals that higher salinity densifies the CO_2_ hydration shell without changing its coordination number, and ions do not accumulate near CO_2_. Hydrogen bond analysis indicates that slower diffusion arises primarily from increased viscosity and steric hindrance from hydrated ions rather than disruption of hydrogen bonds. These molecular-level insights can guide site selection and injection strategy optimization for CO_2_ geological storage in saline aquifers.

## 1. Introduction

Global climate change continues to intensify, and cutting greenhouse gas emissions has become urgent. Consequently, carbon capture, utilization, and storage (CCUS) has emerged as a critical strategy for achieving carbon neutrality [1,2]. The Global CCS Institute reported that the global CO_2_ capture capacity was 416 Mtpa in 2024, more than double the 2017 level. However, the capacity currently in operation remains far below the gigaton-scale level needed by 2050 under the IEA’s Net Zero Emissions Scenario [3,4]. The IPCC Special Report on CCS noted that deep saline aquifers have the largest storage capacity among geological storage options, estimated at 1000–10,000 Gt CO_2_ globally, owing to their wide geographical distribution and substantial storage potential [5,6]. When CO_2_ is injected into saline aquifers, its diffusion behavior directly governs the efficiency of dissolution trapping and affects the long-term safety of CO_2_ storage [7]. Therefore, a comprehensive understanding of CO_2_ diffusion in brine under realistic reservoir conditions is essential for selecting appropriate storage sites, assessing risks, and designing injection strategies in CCUS projects.

Among CO_2_ emission reduction technologies, CCUS has attracted substantial research attention. Before CO_2_ is injected into saline aquifers, the gas captured from industrial sources typically requires compression and purification—processes that incur substantial costs [8]. Izadpanahi et al. recently reviewed all four CO_2_ trapping mechanisms in saline aquifers—structural, residual, dissolution, and mineralization—and concluded that dissolution trapping is the key bridging mechanism that dominates during injection and the early post-injection phase [9]. However, natural saline aquifers typically contain ions such as Na^+^ and Cl^−^, and these ions can significantly alter the microscopic structure and physical properties of water, thereby affecting CO_2_ transport and diffusion [10]. Systematically characterizing CO_2_ diffusion across a range of salinity conditions is essential for assessing CO_2_ migration behavior and long-term trapping efficiency in realistic geological formations.

Dissolution trapping in saline aquifers involves several physical and chemical steps: CO_2_ dissolves at the gas-brine boundary, molecules diffuse through the boundary layer, and density differences in the brine cause convective mixing. Among these, molecular diffusion is the rate-limiting step that controls the long-term dissolution rate after the early convection dies down [11]. In recent years, molecular dynamics (MD) simulation has become a powerful tool for understanding transport mechanisms at the atomic scale. Zhang et al. reviewed microscopic simulation methods for CO_2_–water–rock systems [12]. They noted that MD can capture molecular interaction details beyond the reach of continuum models, including ion-specific hydration, hydrogen bond dynamics, and local structural ordering around dissolved gases. Coelho et al. studied the Soret effect of CO_2_ in NaCl brines using non-equilibrium MD [13]. They found that as temperature rises, CO_2_ switches from thermophobic to thermophilic behavior. This change is mainly attributed to alterations in the water hydrogen bond network, rather than direct ion–CO_2_ interactions. These findings demonstrate that molecular-level work is essential for building reliable predictive models of CO_2_ transport in saline aquifers.

The composition of saline aquifers and the corresponding CO_2_ diffusion behavior significantly influence storage performance. Saline aquifers are predominantly composed of water with dissolved ions such as Na^+^ and Cl^−^. CO_2_ diffusion in this water is driven by concentration gradients, but it is also modulated by temperature and pressure [14]. Zhang et al. used field-scale simulations and showed that brine salinity is a key factor controlling the relative contributions of dissolution trapping and residual trapping. Higher salinity reduces CO_2_ solubility because of the salting-out effect [15]. Previous work by Carroll et al. demonstrated that salt ions generally reduce CO_2_ solubility in water—this is the well-documented salting-out effect [16]. Chen et al. used MD to investigate ion hydration and hydrogen bond structure in NaCl solutions under CO_2_ storage conditions. They found that dissolved CO_2_ disrupts the hydrogen bond network and destabilizes ionic hydration shells [17]. These findings indicate that the salting-out effect is not solely a solubility-related phenomenon—it also has a kinetic dimension that directly affects CO_2_ transport. However, a systematic molecular-level investigation quantifying the combined effects of salt concentration, temperature, and pressure on CO_2_ diffusion remains absent.

Deep saline aquifers offer the largest CO_2_ storage capacity among geological formations, and formation brine is often used directly as the injection fluid [11]. Omrani et al. showed that when salinity increases, the CO_2_ diffusion coefficient decreases, which can retard CO_2_ movement in the reservoir and inhibit dissolution trapping efficiency [18]. Song et al. provided a comprehensive molecular simulation framework for understanding geological carbon storage [19]. This framework covers solubility trapping, interfacial phenomena, and adsorption mechanisms using both MD and Monte Carlo methods. Li et al. recently showed that external physical fields can change CO_2_ dissolution at the molecular scale; for example, electric fields can boost the dissolution rate by up to 42% by reorienting water dipoles at the interface [20]. These findings suggest that a deeper understanding of molecular-scale transport could lead to new engineering strategies for improved CO_2_ storage. Deep saline aquifers are cost-effective and secure options for CO_2_ storage, and the interactions between CO_2_ and formation brine are fundamental to securing long-term storage [21]. Temperature, pressure, and water chemistry all influence CO_2_ diffusion. Changes in salinity affect both ionic strength and water activity, thereby altering the CO_2_ diffusion coefficient. Because salinity varies spatially in natural aquifers and cannot be engineered for storage purposes, systematic studies on CO_2_ diffusion behavior across salinity gradients represent a fundamental research need.

To obtain reliable diffusion data, a CO_2_–brine model that matches real saline aquifer conditions must be constructed, and MD simulations must be run at realistic temperatures and pressures. We performed MD simulations spanning NaCl concentrations of 0.1–5.0 mol/L, temperatures of 298–353 K, and pressures of 3–40 MPa. Diffusion coefficients were derived from MSD, and RDF combined with hydrogen bond analyses was used to resolve the underlying molecular mechanisms. The results quantify the competing effects of salinity, temperature, and pressure on CO_2_ diffusivity and identify viscosity enhancement—rather than hydrogen bond disruption—as the primary mechanism by which salt ions retard CO_2_ transport.

## 2. Results and Discussion

### 2.1. CO_2_ Diffusion Coefficients Under Varying Salinity, Temperature, and Pressure

**Figure 1:**
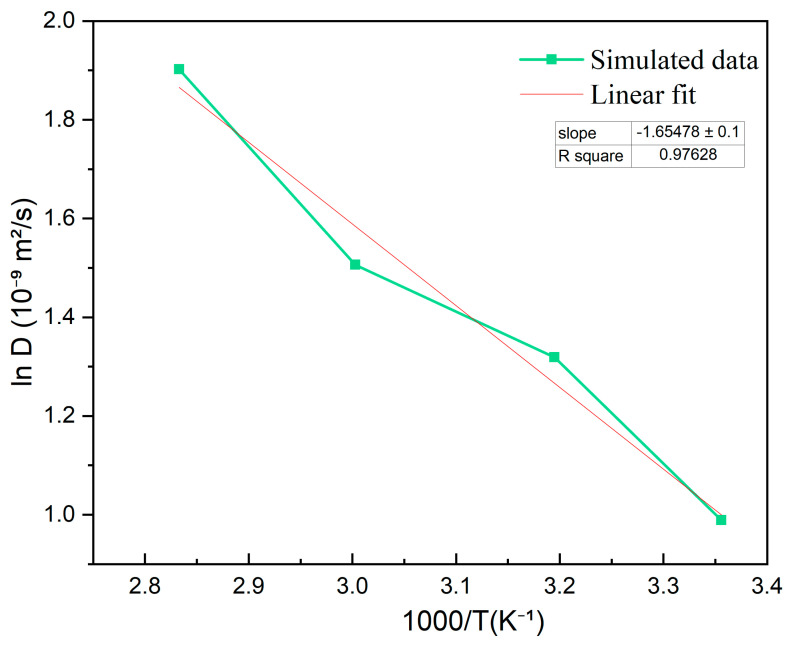
Arrhenius plot of the CO_2_ diffusion coefficient in brine at 3.5 mol/L NaCl and 10 MPa. The linear fit yields an activation energy of 13.8 kJ/mol.

**Figure 2:**
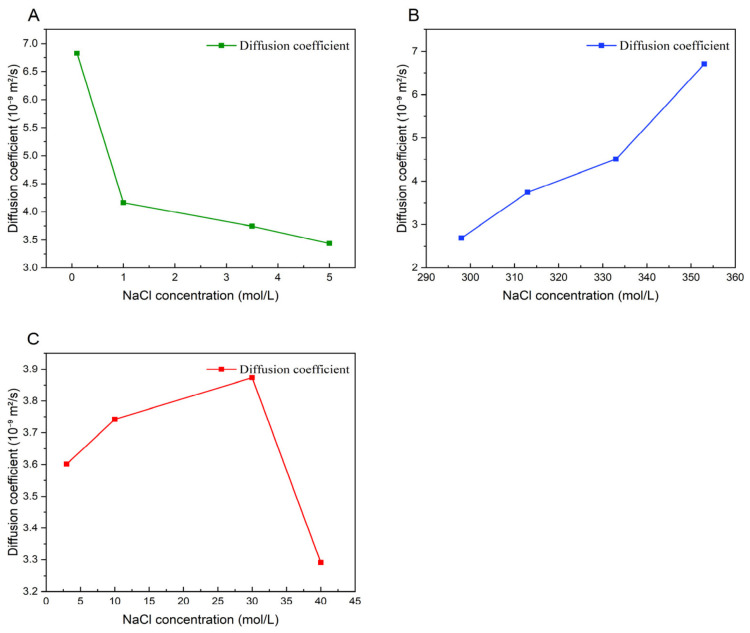
Diffusion coefficient of CO_2_ in brine as a function of (**A**) NaCl concentration, (**B**) temperature, and (**C**) pressure.

The CO_2_ diffusion coefficients in brine under different salinity, temperature, and pressure conditions were obtained from the MD simulations. By analyzing the MSD, we compared and quantified the influence of these three factors on CO_2_ diffusion. Figure 1 presents the Arrhenius plot confirming the thermally activated nature of CO_2_ diffusion, while Figure 2 provides a comprehensive overview of the diffusion coefficient as a function of all three variables.

#### 2.1.1. Influence of Salinity

Figure 2A plots the CO_2_ diffusion coefficient against NaCl concentration at 313 K and 10 MPa. As salinity increases from 0.1 to 5.0 mol/L, coefficient D decreases by approximately 49.6% (from 6.83 × 10^−9^ to 3.44 × 10^−9^ m^2^/s), consistent with the well-documented salting-out effect [16]. The decline is more pronounced at low concentrations (0.1 → 1.0 mol/L accounts for ~39% of the total decrease) and attenuates at higher concentrations (3.5 → 5.0 mol/L accounts for only ~8%). This non-linear behavior arises because ionic hydration shells gradually saturate. At low salinity, added ions hydrate strongly and sequester free water molecules, which rapidly increases the solution viscosity and retards CO_2_ diffusion. As concentration increases, the hydration shells are nearly saturated, and therefore adding more ions has a diminished effect on the solution structure. The RDF analysis in Section 2.2.1 supports this: the first hydration shell of CO_2_ becomes denser (higher peak, narrower FWHM) with salinity, but it does not incorporate more water molecules. The hydrogen bond analysis in Section 2.3 provides further support: the CO_2_–water hydrogen bond count decreases by only ~5% over the same salinity range. The dominant mechanism is therefore increased solution viscosity rather than direct ion blocking.

#### 2.1.2. Influence of Temperature

Figure 1 shows that ln D and 1000/T have a clear linear relationship at 3.5 mol/L NaCl and 10 MPa, indicating that CO_2_ diffusion follows Arrhenius behavior. The activation energy obtained from the fit is 13.8 kJ/mol, which is well within the range of 13.5–16.2 kJ/mol that Omrani et al. [18] reported under similar salinity, supporting the consistency of the present results. Figure 2B shows the corresponding temperature trend: when the temperature increases from 298 to 353 K, coefficient D increases from 2.69 × 10^−9^ to 6.70 × 10^−9^ m^2^/s—an increase of approximately 149%, consistent with the enhanced molecular mobility at elevated temperatures [18].

At the molecular level, E_a_ is the energy barrier CO_2_ molecules must overcome to escape from the surrounding water and ions and make a diffusive jump. The activation energy obtained is higher than the typical value for CO_2_ in pure water, which indicates that salt ions impede diffusion. However, temperature enhances diffusion to a greater extent than salinity suppresses it. As described in Section 2.2, higher temperature disrupts the ordered hydration shell (the RDF peak decreases in height and broadens), reduces local viscosity and lowers the barrier for CO_2_ to escape from its hydration cage. Even at the high salinity of 3.5 mol/L NaCl, the CO_2_ diffusion coefficient at 353 K (6.70 × 10^−9^ m^2^/s) remains substantially higher than the value of 3.08 × 10^−9^ m^2^/s reported by Perera et al. in pure water at 323 K [22]. It should be noted that the picture of CO_2_ molecules making discrete diffusive jumps by escaping their hydration cages is an interpretation based on the observed Arrhenius behavior, rather than on direct visualization of individual molecular trajectories. An alternative mechanism—in which CO_2_ moves within a partially retained hydration shell without a distinct cage-escape event—cannot be ruled out by the present MSD- and RDF-based analyses. Future studies employing residence-time analysis of water molecules in the CO_2_ hydration shell would help to distinguish these two microscopic pictures. Nevertheless, regardless of the detailed mechanism, the pronounced temperature sensitivity confirms the dominant role of thermal activation in CO_2_ transport. In the temperature range investigated, temperature is the dominant factor controlling CO_2_ transport in saline aquifers.

#### 2.1.3. Influence of Pressure

Figure 2C shows how the CO_2_ diffusion coefficient changes with pressure at 3.5 mol/L NaCl and 313 K. Between 3 and 30 MPa, D increases slightly from 3.60 × 10^−9^ to 3.87 × 10^−9^ m^2^/s (approximately 7.5%). This is consistent with other studies reporting that pressure exerts only a minor influence on CO_2_ diffusion under typical storage conditions [18]. The weak increase occurs because the solution density varies only slightly in this pressure range, so the free volume available for molecular displacement does not change significantly.

However, when the pressure increases to 40 MPa, D decreases markedly to 3.29 × 10^−9^ m^2^/s—approximately 15% lower than at 30 MPa. This observation suggests that at pressures above ~30 MPa, the reduction in free volume may begin to outweigh any facilitating effects on molecular displacement, though additional data points between 30 and 40 MPa would be needed to confirm this trend. Above about 30 MPa, the solution density becomes sufficiently high to outweigh any facilitating effects on molecular movement. The denser solution reduces free volume and increases friction, and CO_2_ diffusion is retarded. This indicates that in very deep formations (deeper than ~2.5–3 km, assuming a geobaric gradient of ~10 MPa/km), pressure may hinder CO_2_ movement, suggesting that the conventional view—that pressure is a secondary factor for CO_2_ diffusion—may require qualification for ultra-deep reservoirs. Overall, in the typical storage pressure range (10–30 MPa), pressure is not the primary controlling factor for CO_2_ diffusion. However, the threshold identified at 40 MPa suggests that caution should be exercised when using diffusion data for ultra-deep saline aquifers.

**Figure 3:**
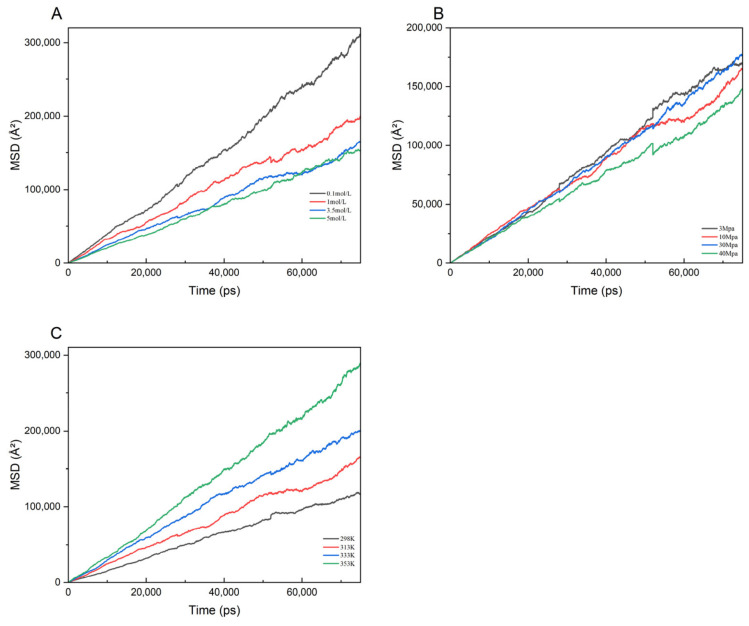
Mean square displacement (MSD) of CO_2_ molecules in brine under different conditions. (**A**) Varying NaCl concentrations at 313 K and 10 MPa; (**B**) varying temperatures at 3.5 mol/L NaCl and 10 MPa; (**C**) varying pressures at 3.5 mol/L NaCl and 313 K.

To verify the reliability of the diffusion coefficients, the MSD curves for representative conditions are shown in Figure 3. The linear region employed for fitting (10–50 ns) yielded R^2^ > 0.99, confirming that the Einstein relation is satisfied.

### 2.2. Radial Distribution Function Analysis of CO_2_ with Water and Ions

To investigate how salt concentration, temperature, and different ions affect the local hydration structure around CO_2_, the radial distribution functions g(r) were calculated between CO_2_ and water oxygen, Na^+^, and Cl^−^. By examining the first peak position, peak height, and coordination number, the local arrangement of water molecules around CO_2_ and the influence of ions on this structure were characterized.

#### 2.2.1. Influence of Salinity on the CO_2_–Water RDF

**Figure 4:**
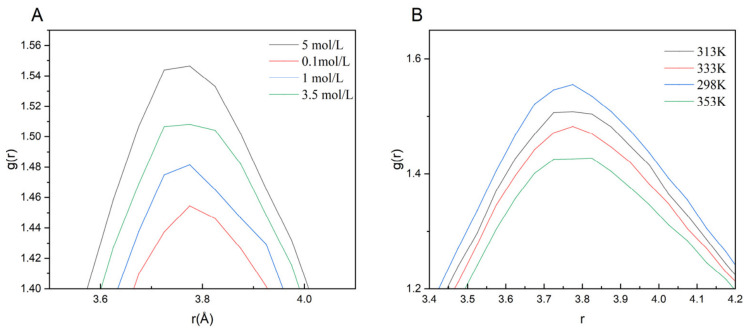
Radial distribution functions of CO_2_ in brine. (**A**) CO_2_–O(H_2_O) RDF at different NaCl concentrations (313 K, 10 MPa); (**B**) CO_2_–O(H_2_O) RDF at different temperatures (3.5 mol/L NaCl, 10 MPa).

Figure 4A shows that when NaCl concentration increases from 0.1 to 5.0 mol/L, the first peak of the CO_2_–O(H_2_O) RDF increases (from 1.45 to 1.55), but the peak position remains at approximately 3.8 Å. A higher peak indicates water molecules are packed more densely around CO_2_, forming a more ordered local structure. From the coordination numbers in Table 1, the results indicate that the number of water molecules in the first hydration shell remains nearly constant at approximately 4.0. Thus, the shell becomes denser through compression, rather than through incorporation of additional water molecules. This occurs because ions compete for water: Na^+^ and Cl^−^ form strong hydration shells that consume substantial free water, and the remaining water is confined more tightly around CO_2_. A denser hydration shell hinders CO_2_ mobility, which is consistent with the decrease in diffusion coefficient with salinity reported in Section 2.1.

#### 2.2.2. Influence of Temperature on the CO_2_–Water RDF

Figure 4B shows that as temperature increases from 298 to 353 K, the first CO_2_–O(H_2_O) RDF peak decreases and broadens, and the peak position shifts slightly from about 3.7 to 3.8 Å. These changes indicate that stronger thermal motion disrupts the ordered water structure around CO_2_, resulting in a more uniform local distribution. At 298 K, water molecules exhibit less thermal motion and can form a tighter hydration layer around CO_2_, yielding a higher g(r) peak. This microstructural change explains why the diffusion coefficient increases significantly with temperature in Section 2.1: thermal energy weakens the hydration cage, thereby enhancing CO_2_ mobility.

#### 2.2.3. CO_2_–Ion Radial Distribution Functions

**Figure 5:**
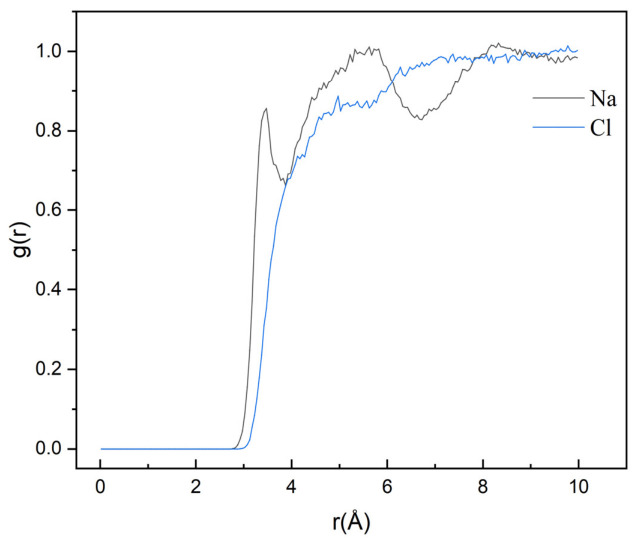
CO_2_–Na^+^ and CO_2_–Cl^−^ RDF at 3.5 mol/L NaCl (313 K, 10 MPa).

Figure 5 compares the RDFs of CO_2_–Na^+^ and CO_2_–Cl^−^ at 3.5 mol/L NaCl. CO_2_–Na^+^ shows a clear peak at about 3.3 Å (g(r) ≈ 1.18) and a shallow dip near 4.0 Å. CO_2_–Cl^−^, in contrast, exhibits no distinct peaks and remains near 1.0 over the entire range. This difference arises from the charge distribution in CO_2_. Na^+^ is attracted to the negatively charged oxygen atoms of CO_2_, but Cl^−^ is repelled by those same negative charges, and Cl^−^ is further separated from CO_2_ by its own strongly bound hydration shell. The CO_2_–Na^+^ peak is much lower than typical ion–water RDF peaks, indicating that this interaction is weak and transient. Therefore, direct blocking of CO_2_ by ions is negligible, and the primary mechanism by which higher salinity retards diffusion remains the increase in solution viscosity and the densification of the CO_2_ hydration shell.

**Figure 6:**
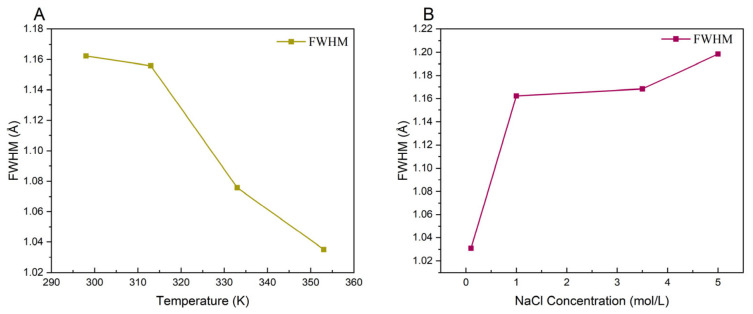
Full width at half maximum (FWHM) of the first CO_2_–O(H_2_O) RDF peak as a function of (**A**) NaCl concentration and (**B**) temperature.

To examine more closely the ordering of the first CO_2_ hydration shell, the full width at half maximum (FWHM) of the first CO_2_–O(H_2_O) RDF peak was calculated. The results are presented in Figure 6. When NaCl concentration increases from 0.1 to 5.0 mol/L, the FWHM decreases slightly from approximately 1.19 to 1.17 Å, indicating that the hydration shell becomes marginally narrower. Together with the higher peak (Figure 4A) and the nearly constant coordination number (Table 1), this confirms that the shell becomes denser rather than incorporating more water molecules. In contrast, when the temperature is raised from 298 to 353 K, the FWHM increases from about 1.10 to 1.24 Å, indicating pronounced thermal broadening. This is consistent with the lower peak in Figure 4B and directly reflects thermal disruption of the ordered hydration structure. The total FWHM change is within ~0.15 Å across all conditions, indicating that the first CO_2_ hydration shell is structurally robust. The opposite FWHM responses to salinity and temperature provide a clear structural explanation for why the diffusion coefficient varies in opposite directions: a denser shell retards diffusion, while thermal broadening enhances it.

### 2.3. Changes in the Hydrogen Bond Network and the Role of Salt Ions

**Figure 7:**
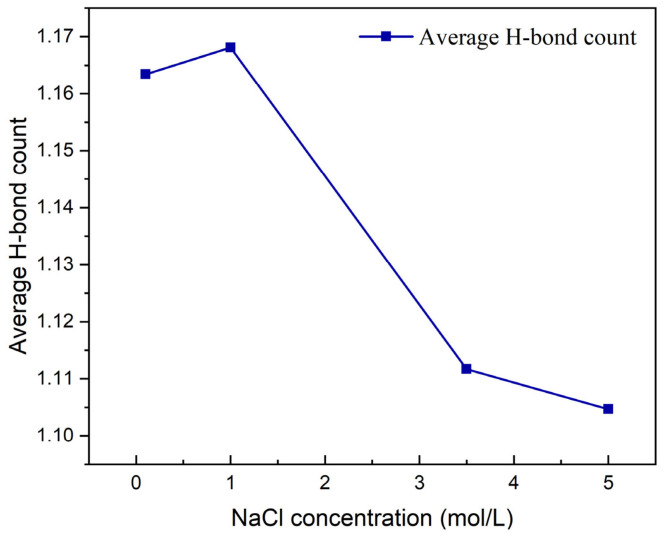
Average number of hydrogen bonds per CO_2_ molecule with water at different NaCl concentrations.

The extent to which salt ions perturb CO_2_–water interactions was quantified through hydrogen bond analysis. The average number of hydrogen bonds per CO_2_ molecule was calculated at different NaCl concentrations. A hydrogen bond was defined as O···H distance < 2.5 Å and O–H···O angle > 120°. The simulation system contained 53 CO_2_ molecules, and Figure 7 shows the average hydrogen bond count per CO_2_. When the NaCl concentration was increased from 0.1 to 5.0 mol/L, the average count decreased slightly, from 1.163 to 1.105—a decrease of approximately 5%. (At 0.1 and 1.0 mol/L, the values overlapped within error.) This trend is consistent with the coordination numbers in Table 1 and shows that salt ions exert minimal influence on the local hydration around CO_2_.

A reduction in hydrogen bonds might superficially imply weaker attraction between CO_2_ and water, which would allow CO_2_ to diffuse faster. However, the diffusion coefficient decreases by approximately 50% as salinity increases. This marked contrast provides strong evidence that slower diffusion at high salinity is not caused by hydrogen bond disruption. Instead, it is primarily attributable to a significant increase in solution viscosity and steric hindrance from hydrated ions. At high salinity, ionic hydration shells start to overlap. This reduces the proportion of free water and increases the bulk viscosity, and increases the resistance to CO_2_ motion.

**Figure 8:**
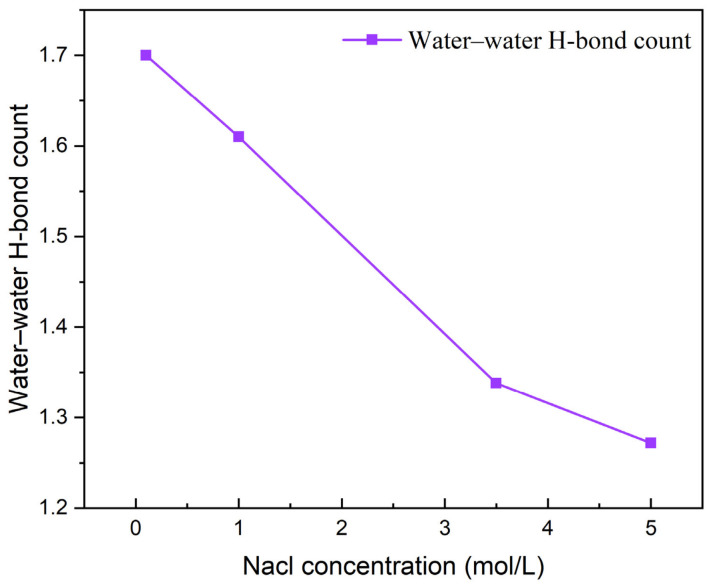
Average number of water–water hydrogen bonds per water molecule as a function of NaCl concentration at 313 K and 10 MPa.

While the CO_2_–water hydrogen bond count remains nearly constant across the full salinity range, the water–water hydrogen bond network undergoes pronounced disruption. Figure 8 shows the average number of water–water hydrogen bonds per water molecule as a function of NaCl concentration. As the salinity increases from 0.1 to 5.0 mol/L, this number decreases substantially from 1.70 to 1.27, a reduction of approximately 25%. This decline reflects the progressive withdrawal of water molecules from the bulk hydrogen bond network into the hydration shells of Na^+^ and Cl^−^, consistent with the formation of contact ion pairs that sequester free water and elevate the solution viscosity.

**Figure 9:**
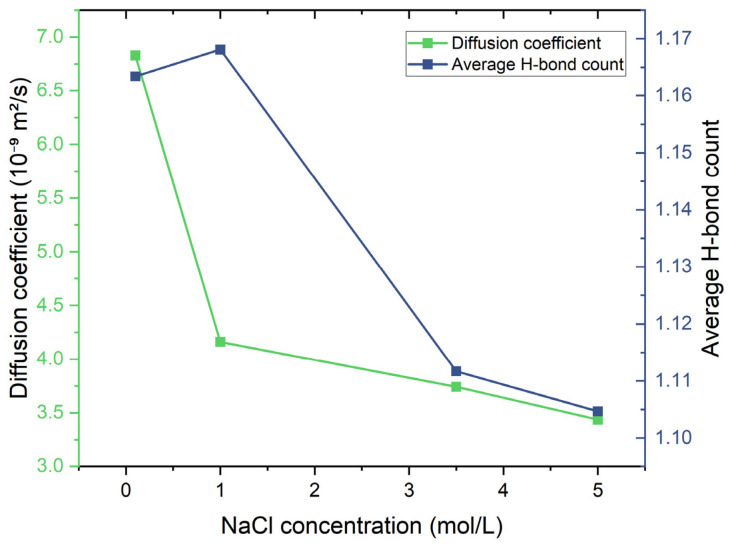
Comparison of the average number of hydrogen bonds per CO_2_ molecule (left axis) and the CO_2_ diffusion coefficient (right axis) as a function of NaCl concentration at 313 K and 10 MPa.

Figure 9 illustrates this contrast clearly: when NaCl increases from 0.1 to 5.0 mol/L, hydrogen bonds per CO_2_ decrease by only ~5%, but the diffusion coefficient decreases by ~50%. This mismatch, together with the concurrent disruption of the water–water hydrogen bond network (Figure 8), provides convergent evidence that salt ions retard CO_2_ diffusion primarily through elevated solution viscosity and steric hindrance from hydrated ions, rather than through disruption of CO_2_–water hydrogen bonds. It should be noted that the ~5% decrease in CO_2_–water hydrogen bonds is based on a geometric definition (O···H < 2.5 Å, O–H···O > 120°), and fluctuations in hydrogen bond populations are inherent to MD simulations. The absolute hydrogen bond counts should therefore be interpreted with this sensitivity in mind; nevertheless, the qualitative conclusion—that the change in hydrogen bonding is far smaller than the change in diffusion—is robust regardless of the specific cutoff values.

### 2.4. Comparison with Literature Results and Validation of Model Reliability

The simulation methodology was validated by comparing the CO_2_ diffusion coefficients with published experimental and MD data. Polat et al. [23] reported a comprehensive review of both experiments and simulations for CO_2_ diffusion in water.

Across the salinity range, the ~50% decline in D from 0.1 to 5.0 mol/L NaCl at 313 K and 10 MPa aligns with previous reports. Omrani et al. [18] found D reductions of 15–64% over 1–6 mol/kg NaCl from MD simulations; Cadogan et al. [24] confirmed the same trend using ^13^C NMR at 298 K; and Perera et al. [22] measured a ~45% decrease in 4 mol/L NaCl by Raman spectroscopy. The quantitative differences among studies are modest and expected, given variations in force field parameterization, concentration units, and experimental conditions. What is consistent across all studies is the underlying mechanism: ions sequester free water into hydration shells (reflected in the 25% loss of water–water hydrogen bonds in Section 2.3), which raises bulk viscosity, while the CO_2_ hydration shell simultaneously densifies without adding water molecules (higher g(r) peak, narrower FWHM, constant coordination number ≈ 4.0).

The temperature dependence also agrees well with the literature. The present activation energy of 13.8 kJ/mol falls within the 13.5–16.2 kJ/mol range reported by Omrani et al. [18]. This consistency across force fields suggests that the energy barrier for CO_2_ diffusive jumps is fundamentally set by the reorganization of the surrounding hydrogen bond network. The RDF and FWHM analyses (Section 2.2.2) support this interpretation: elevated temperature broadens and lowers the first CO_2_–O(H_2_O) peak, corresponding to thermal disruption of the ordered hydration cage that otherwise constrains CO_2_ mobility.

Pressure effects are minor across 3–30 MPa (D varies by less than 10%), consistent with reports from Sell et al. [25], Perera et al. [22], and Omrani et al. [18]. The 15% drop observed at 40 MPa, however, suggests a threshold beyond which increased solution density and friction outweigh any facilitating contributions from local density fluctuations. This non-monotonic behavior warrants caution when extrapolating diffusion data to ultra-deep reservoirs (depth > ~3 km), where pressure-induced suppression may partially offset the thermal enhancement of CO_2_ mobility.

It is worth noting an apparent tension with Chen et al. [17], who reported that dissolved CO_2_ disrupts the hydrogen bond network and destabilizes ionic hydration shells in NaCl solutions. At first glance, this seems opposite to the present finding that CO_2_–water hydrogen bonds are minimally affected by salinity. The two observations are, however, complementary rather than contradictory. Chen et al. examined how CO_2_ perturbs pre-existing ion–water structures, whereas the present study examines how ions perturb CO_2_–water interactions. The pronounced 25% reduction in water–water hydrogen bonds observed here (Figure 8) is in fact consistent with Chen et al.’s general picture of a disrupted water network—but the present work clarifies that ions, rather than CO_2_, are the primary drivers of this disruption at high salinity. The CO_2_–water hydrogen bond network, being far weaker than ion–water electrostatic interactions, is largely shielded from direct ionic perturbation; instead, it is the bulk reorganization of water into ionic hydration shells that raises the effective viscous resistance to CO_2_ diffusion.

Taken together, the agreement with experimental and computational benchmarks confirms that the COMPASS force field adequately captures the key interactions governing CO_2_ transport in brine. Minor discrepancies in absolute D values are attributable to force field version differences, system size effects on MSD statistics, and experimental uncertainties—none of which affect the mechanistic conclusions. Additionally, the simulation box dimensions (~33 Å per side) are relatively modest for diffusion calculations, and the Yeh–Hummer finite-size correction was not applied in this study. However, because all systems share comparable box dimensions and the analysis focuses on relative trends across conditions rather than absolute D values, the comparative conclusions remain valid.

## 3. Materials and Methods

Molecular dynamics simulation is widely used to study atomic-scale transport. This method was selected for several reasons. First, the COMPASS force field has been specifically parameterized and tested for CO_2_–water–alkali metal halide systems, and it yields reliable estimates for properties such as diffusion coefficients [26]. Second, homogeneous solution systems can be readily constructed with precise control over the amounts of CO_2_, water, and ions, enabling accurate salinity control. Third, the simulation algorithms include thermostats and barostats that maintain temperature and pressure constant, making the method well-suited for studying transport under isothermal–isobaric conditions. The parameters of the constructed systems are shown in Table 2.

### 3.1. Model Structure

**Figure 10:**
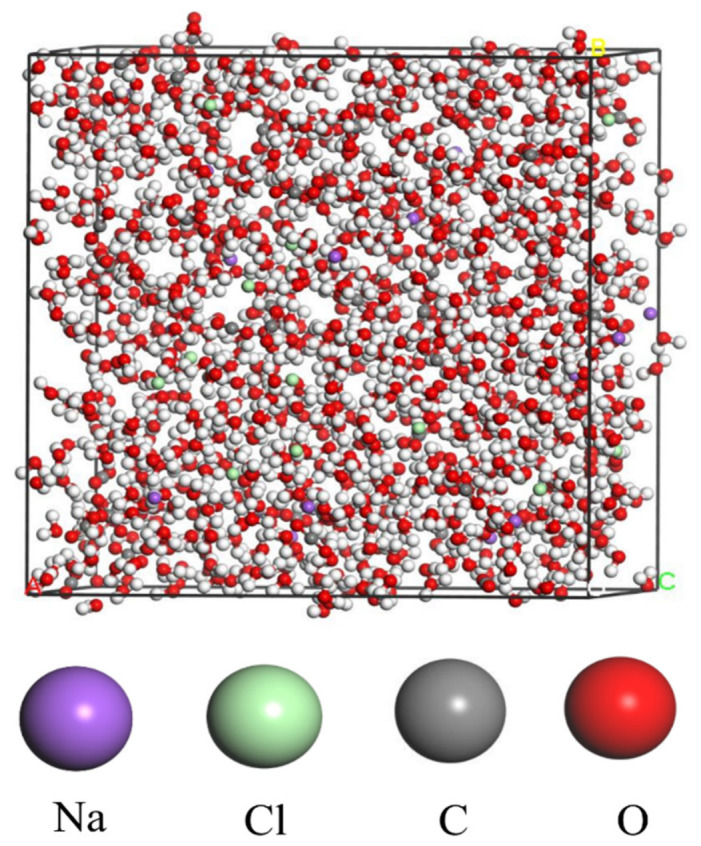
Schematic representation of the molecular dynamics model for CO_2_ in NaCl brine (1.0 mol/L).

The brine system was constructed from water, CO_2_, and NaCl. The initial three-dimensional structures of H_2_O and CO_2_ were obtained from the PubChem database [27,28], and standard ion models were adopted for Na^+^ and Cl^−^. Four systems were constructed with NaCl concentrations of 0.1, 1.0, 3.5, and 5.0 mol/L. Each system initially contained 1000 H_2_O molecules and 53 CO_2_ molecules, and the numbers of Na^+^–Cl^−^ ion pairs were 2, 18, 73, and 90. These values were determined from the target salt concentrations and the CO_2_ mole fraction. Initial box dimensions were estimated from the total number of molecules and the density of pure water at 313 K. After building the systems, energy minimization was performed to remove unfavorable atomic overlaps and relieve high-energy strain, with stringent convergence criteria (energy change < 1 × 10^−5^ kcal/mol; maximum force < 0.001 kcal/mol/Å). A short NVT equilibration was then performed, followed by an NPT equilibration at 313 K and 0.1 MPa to obtain realistic equilibrium densities. Table 1 lists the final system parameters after the NPT equilibration, and Figure 10 shows the constructed model.

### 3.2. Force Field and Parameters

The accuracy of molecular simulation depends on the force field. All simulations were performed using a commercial MD simulation platform. The COMPASS force field describes all interatomic interactions, with atomic charges assigned automatically. COMPASS has been specifically developed for modeling CO_2_, water, and alkali metal halide solutions, and it has been widely tested for predicting transport properties such as density and diffusion coefficients in liquid mixtures [26]. It accurately captures CO_2_–water, ion–water, and ion–CO_2_ non-bonded interactions, enabling reliable simulation of CO_2_ diffusion in brine.

Non-bonded interactions were treated as follows: electrostatic interactions were calculated with the Ewald summation method (accuracy 0.001 kcal/mol for NVT, 0.0001 kcal/mol for NPT, buffer width 0.5 Å). This study used the Lennard-Jones potential with an atom-based summation and cubic spline cutoff to model van der Waals interactions. Cutoff distance was set to 15.5 Å, the spline width to 1 Å, and long-range corrections were applied.

### 3.3. Simulation Details

The simulations were conducted in two stages: first, NPT equilibration; then, NVT production. During the NPT, the temperature was maintained at 313 K with a Nosé–Hoover thermostat (time constant = 0.1 ps) [29] and the pressure at 0.01 GPa (10 MPa) with a Berendsen barostat (pressure coupling time constant = 0.1 ps) [30]. A time step of 1.5 fs was used, and the equilibration was run for 1000 ps (approximately 666,667 steps).

After the NPT, NVT production runs of 100 ns were performed. Temperature was controlled using a Nosé–Hoover thermostat. The time step was 2.0 fs, yielding 50,000,000 steps. O–H bonds were constrained using the SHAKE algorithm, enabling a time step of 2.0 fs. To investigate the effects of salinity, temperature, and pressure on CO_2_ diffusion, we designed a series of NVT runs using the controlled-variable method. The NaCl concentrations were set to 0.1, 1.0, 3.5, and 5.0 mol/L, the temperature ranged from 298 to 353 K, and the pressure from 3 to 40 MPa. Table 3 lists all the simulation conditions. All reported quantities were averaged over the final 50 ns of each NVT run, after confirming that the system had reached equilibrium.

### 3.4. Diffusion Coefficient and Microscopic Analysis Methods

The tracer diffusion coefficient of CO_2_ in brine was derived from the mean-square displacement (MSD) using the Einstein equation. MSD analysis was performed using standard modules within the MD platform. The linear region of the MSD curve was fitted, and the slope was substituted into the Einstein equation to obtain the diffusion coefficient D:

(1) $$D=\frac{1}{6}\;\underset{t\to \infty }{lim}\;\frac{d}{dt}\langle {\left|r_{i}(t)-r_{i}(0)\right|}^{2}\rangle$$

where $r_{i}$(t) is the position of the i-th CO_2_ molecule at time t, and the angle brackets mean the average over all CO_2_ molecules and time origins. For some trajectories, the MSD showed non-physical drops at long times because of periodic boundary effects. In those cases, only the linear region before the first drop was used for fitting. The fitted region (10–50 ns) yielded an R^2^ > 0.99. Statistical uncertainties in D were evaluated by block averaging: the final 50 ns of each NVT production run was divided into five 10 ns blocks, the MSD of each block was fitted independently, and the standard deviation of the five resulting D values is reported as the uncertainty in Table S1. The block-averaged uncertainties range from ±6% to ±35%, reflecting the intrinsic statistical fluctuation of CO_2_ diffusion in these moderately sized simulation systems.

To investigate the effect of salt ions on the arrangement of water and ions around CO_2_, the radial distribution functions g(r) were calculated between CO_2_ and H_2_O (oxygen), Na^+^, and Cl^−^. The carbon atom of CO_2_ was used as the reference. The RDF calculations were based on the final 50 ns of each NVT run and used standard RDF analysis routines. The cutoff radius was set to 10.0 Å with a sampling interval of 0.05 Å, and only intermolecular pairs were considered. The position and height of the first peak in the RDF were used to determine the radius of the first CO_2_ hydration shell and the coordination number, as well as to track how the hydration shell changes with salt concentration.

Hydrogen bond analysis was employed to examine how salt ions affect CO_2_–water interactions. If this mechanism dominates, the average number of hydrogen bonds per CO_2_ molecule should decrease substantially with increasing salinity. To evaluate this hypothesis, hydrogen bonds were quantified using a geometric criterion. A hydrogen bond was defined geometrically: an O···H distance of less than 2.5 Å and an O–H···O angle greater than 120°. A custom script was developed to analyze the NVT trajectories frame by frame and calculate the average number of hydrogen bonds per CO_2_ molecule. This analysis enabled an assessment of how salt ions disrupt the CO_2_–water hydrogen bond network.

## 4. Conclusions

In this work, MD simulations were employed to investigate the influence of NaCl concentration on CO_2_ diffusion in saline aquifers, together with the effects of temperature and pressure. Diffusion coefficients were derived from MSD, and RDF and hydrogen bond analysis were used to elucidate the microscopic mechanisms. The main conclusions are as follows:

1. The CO_2_ diffusion coefficient in brine decreases steadily as NaCl concentration increases. At 313 K and 10 MPa, when NaCl increases from 0.1 to 5.0 mol/L, D decreases by approximately 50% (from 6.83 × 10^−9^ to 3.44 × 10^−9^ m^2^/s). This salting-out effect arises from the hydration of Na^+^ and Cl^−^, which increases solution viscosity and densifies the CO_2_ hydration shell.
2. Temperature strongly enhances CO_2_ diffusion. At 3.5 mol/L NaCl and 10 MPa, D increases by approximately 149% from 298 to 353 K (2.69 × 10^−9^ to 6.70 × 10^−9^ m^2^/s), following Arrhenius behavior. Higher temperature increases molecular motion and weakens ion hydration, facilitating CO_2_ migration.
3. Pressure exerts a minor influence on CO_2_ diffusion between 3 and 30 MPa (variation < 8%). However, at 40 MPa, D decreases by approximately 15%, indicating that under very high pressure, the denser solution and reduced free volume begin to suppress diffusion. Under typical storage conditions (10–30 MPa), pressure is not the primary controlling factor, but caution is required for very deep reservoirs.
4. RDF analysis shows that with increasing NaCl concentration, the first CO_2_–O(H_2_O) peak height increases while the coordination number remains around 4.0, indicating that the hydration shell becomes denser rather than larger. CO_2_–Na^+^ exhibits a weak peak near 3.3 Å, whereas CO_2_–Cl^−^ shows no distinct structure; therefore, direct ion blocking is negligible.
5. Hydrogen bond analysis shows that the average number of hydrogen bonds per CO_2_ decreases only slightly (from 1.16 to 1.10) when salinity increases, while the water–water hydrogen bond count per molecule decreases substantially from 1.70 to 1.27. This contrast demonstrates that salt ions retard CO_2_ diffusion primarily through increased viscosity and steric hindrance from hydrated ions, rather than by disrupting CO_2_–water hydrogen bonds.

## Figures and Tables

**Table 1:** Coordination number of the first hydration shell of CO_2_ at different NaCl concentrations.

NaCl Conc. (mol/L)	First Peak Position	First Peak Height	Coordination Number
0.1	3.775	1.45452194	3.99472022
1	3.775	1.48152024	4.10263477
3.5	3.775	1.50814863	3.94462619
5	3.775	1.54645008	3.99684815

**Table 2:** Simulation System Parameter Table.

System	NaCl Conc. (mol/L)	H_2_O Molecules	Na^+^/Cl^−^ Pairs	CO_2_ Molecules	Box Size (Å^3^)
S0	0.1	1000	2	53	32.70 × 32.70 × 32.70
S1	1	1000	18	53	32.73 × 32.73 × 32.73
S2	3.5	1000	73	53	33.44 × 33.44 × 33.44
S3	5	1000	90	53	33.60 × 33.60 × 33.60

**Table 3:** Summary of simulation cases for NVT production runs in this study.

Case	NaCl Conc. (mol/L)	Temperature (K)	Pressure (MPa)	Purpose
1	3.5	313	10	Baseline
2	0.1	313	10	Salinity effect
3	1	313	10	Salinity effect
4	5	313	10	Salinity effect
5	3.5	298	10	Temperature effect
6	3.5	333	10	Temperature effect
7	3.5	353	10	Temperature effect
8	3.5	313	3	Pressure effect
9	3.5	313	30	Pressure effect
10	3.5	313	40	Pressure effect

## Data Availability

The original contributions presented in this study are included in the article/Supplementary Materials. Further inquiries can be directed to the corresponding author.

## Supplementary Materials

The following supporting information can be downloaded at: https://www.mdpi.com/article/10.3390/molecules31122043/s1, Figure S1: Mean square displacement (MSD) curves of CO_2_ at different NaCl concentrations (313 K, 10 MPa); Figure S2: Representative MSD curve with linear fitting for CO_2_ at 3.5 mol/L NaCl, 313 K and 10 MPa (R^2^ > 0.99); Figure S3: MSD curves of CO_2_ at different pressures (3.5 mol/L NaCl, 313 K); Figure S4: MSD curves of CO_2_ at different temperatures (3.5 mol/L NaCl, 10 MPa); Figure S5: Radial distribution functions g(r) for CO_2_–O(H_2_O) at different temperatures (3.5 mol/L NaCl, 10 MPa); Figure S6: Radial distribution functions g(r) for CO_2_–O(H_2_O) at different NaCl concentrations (313 K, 10 MPa); Table S1: Detailed diffusion coefficients of CO_2_ under all simulated conditions with standard deviations (salinity, temperature, pressure) with standard deviations.
