# Correction: Zhou, T.; Li, D. Molecular-Level Insights into CO_2_ Dissolution Trapping in Deep Saline Aquifers: Diffusion Behavior in NaCl Brines. *Molecules* 2026, *31*, 2043

**DOI:** 10.3390/molecules31193497

**Published:** 2026-10-01

**Authors:** Tiankuo Zhou, Dexiang Li

**Affiliations:** 1 SDU-ANU Joint Science College, Shandong University, Weihai 264209, China; 202300700155@mail.sdu.edu.cn; 2 School of Nuclear Science, Energy and Power Engineering, Shandong University, Jinan 250061, China

**Error in Figures**

In the original publication [1], there were errors in the x-axis labels in Figures 2 and 4. The x-axes in Figure 2B,C were incorrectly labeled “NaCl concentration (mol/L)”, and the x-axis in Figure 4B was incorrectly labeled “r”. The corrected Figure 2 and Figure 4 appear below. The authors state that the scientific conclusions are unaffected. This correction was approved by the Academic Editor. The original publication has also been updated.

## Figures and Tables

**Figure 2:**
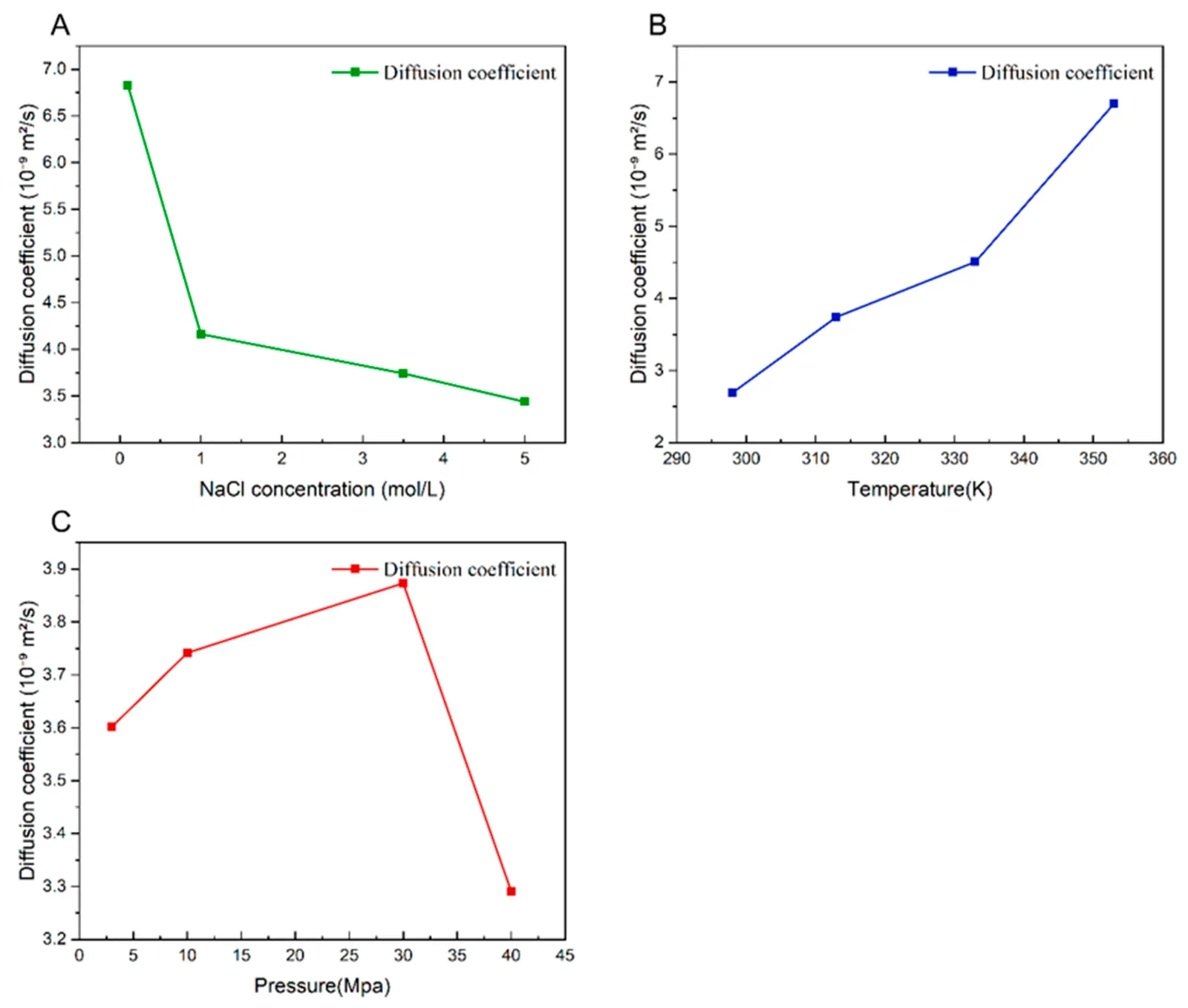
Diffusion coefficient of CO_2_ in brine as a function of (**A**) NaCl concentration, (**B**) temperature, and (**C**) pressure.

**Figure 4:**
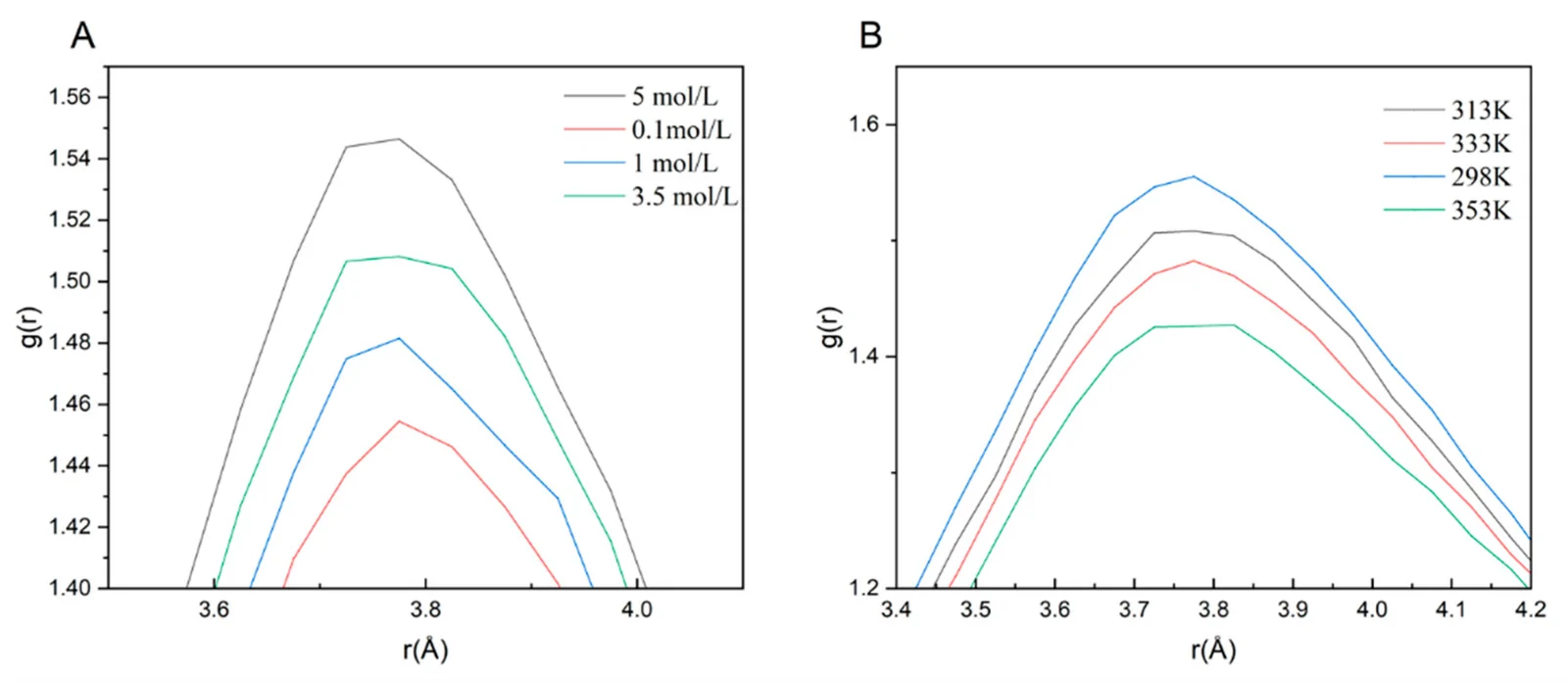
Radial distribution functions of CO_2_ in brine. (**A**) CO_2_–O(H_2_O) RDF at different NaCl concentrations (313 K, 10 MPa); (**B**) CO_2_–O(H_2_O) RDF at different temperatures (3.5 mol/L NaCl, 10 MPa).
