# Supplementary material for: Molecular-Level Insights into CO2 Dissolution Trapping in Deep Saline Aquifers: Diffusion Behavior in NaCl Brines
Source: Molecules. 2026 Jun 11;31(12):2043. doi: 10.3390/molecules31122043 (PMC13304922; doi:10.3390/molecules31122043)
Supplement: Supplementary file 1 [file molecules-31-02043-s001.zip › molecules-4360996-supplementary.pdf]

Supporting Information for

Molecular-Level Insights into CO_2 Dissolution Trapping in Deep Saline Aquifers: Diffusion Behavior in NaCl Brines

Content

Figure S1. Mean square displacement (MSD) curves of CO_2 at different NaCl concentrations (313 K, 10 MPa).

Figure S2. Representative MSD curve with linear fitting for CO_2 at 3.5 mol/L NaCl, 313 K and 10 MPa ($R^2 > 0.99$).

Figure S3. Mean square displacement (MSD) curves of CO_2 at different pressures (3.5 mol/L NaCl, 313 K).

Figure S4. Mean square displacement (MSD) curves of CO_2 at different temperatures (3.5 mol/L NaCl, 10 MPa).

Figure S5. Radial distribution functions g(r) for CO_2–O(H_2O) at different temperatures (3.5 mol/L NaCl, 10 MPa).

Figure S6. Radial distribution functions g(r) for CO_2–O(H_2O) at different NaCl concentrations (313 K, 10 MPa).

Table S1. Detailed diffusion coefficients of CO_2 under all simulated conditions (salinity, temperature, pressure) with standard deviations.

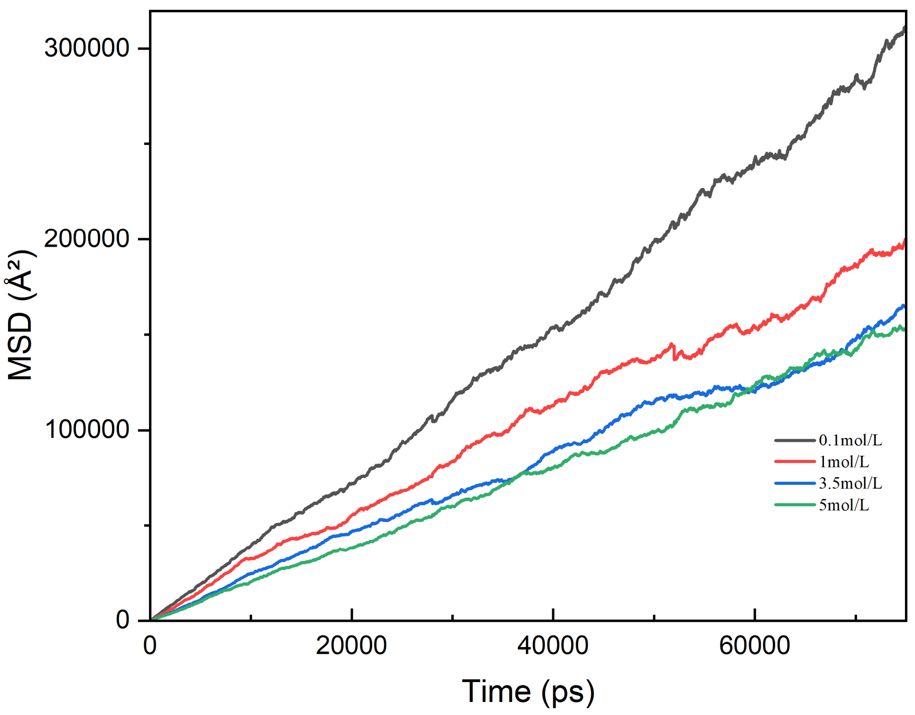

Figure S1. Mean square displacement (MSD) curves of CO_2 at different NaCl concentrations (313 K, 10 MPa).

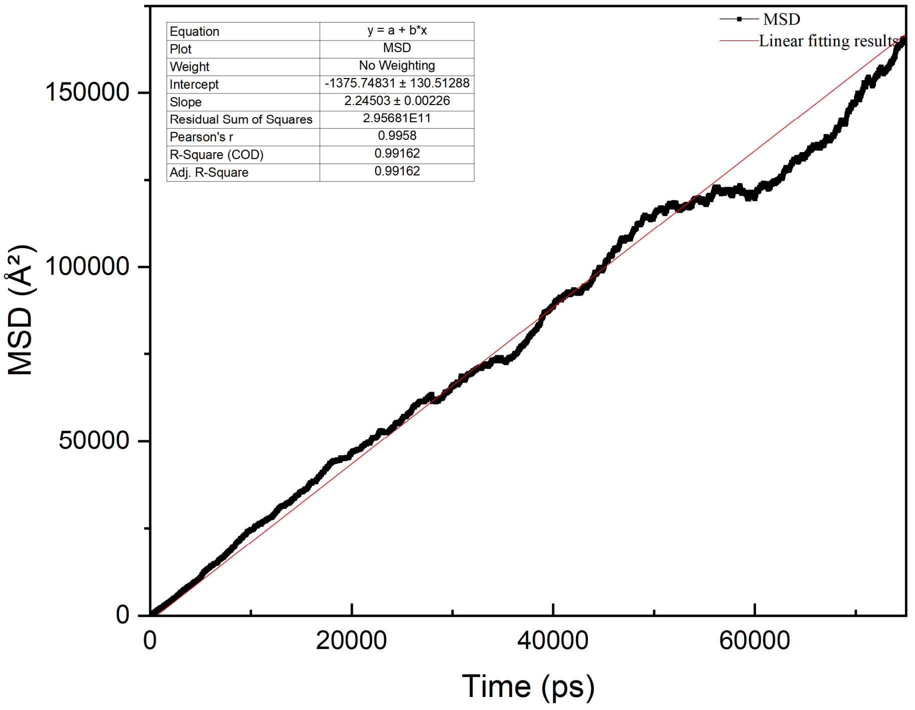

Figure S2. Representative MSD curve with linear fitting for CO_2 at 3.5 mol/L NaCl, 313 K and 10 MPa ($R^2 > 0.99$).

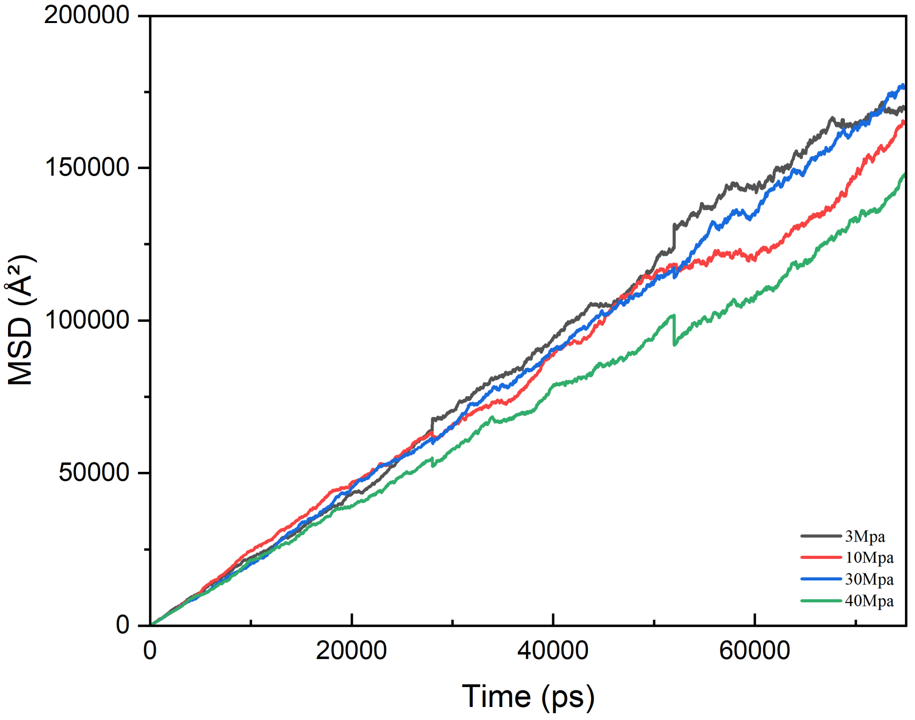

Figure S3. Mean square displacement (MSD) curves of CO_2 at different pressures (3.5 mol/L NaCl, 313 K).

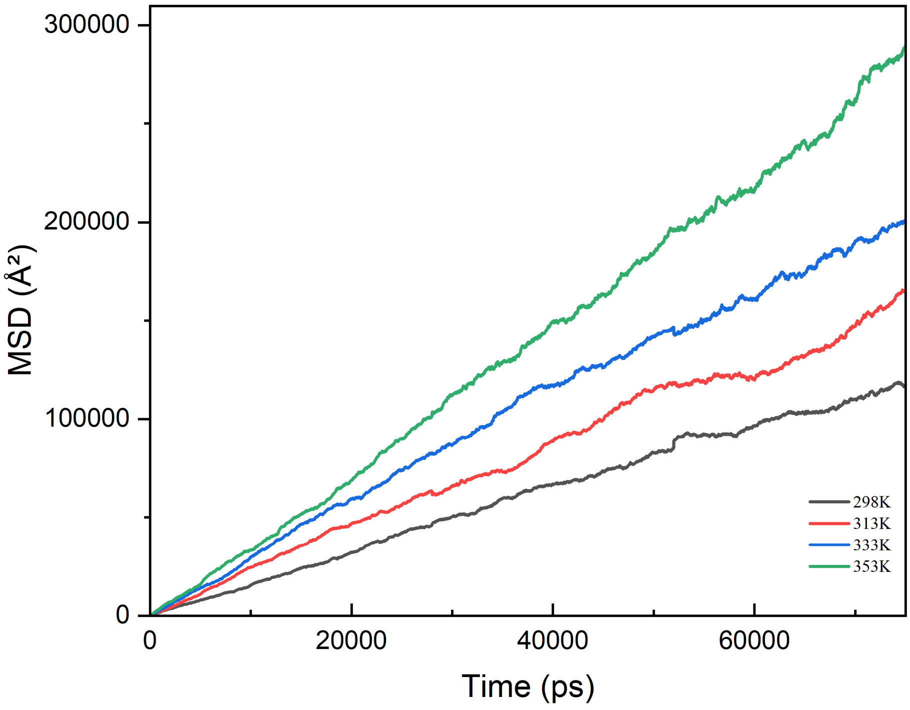

Figure S4. Mean square displacement (MSD) curves of CO_2 at different temperatures (3.5 mol/L NaCl, 10 MPa).

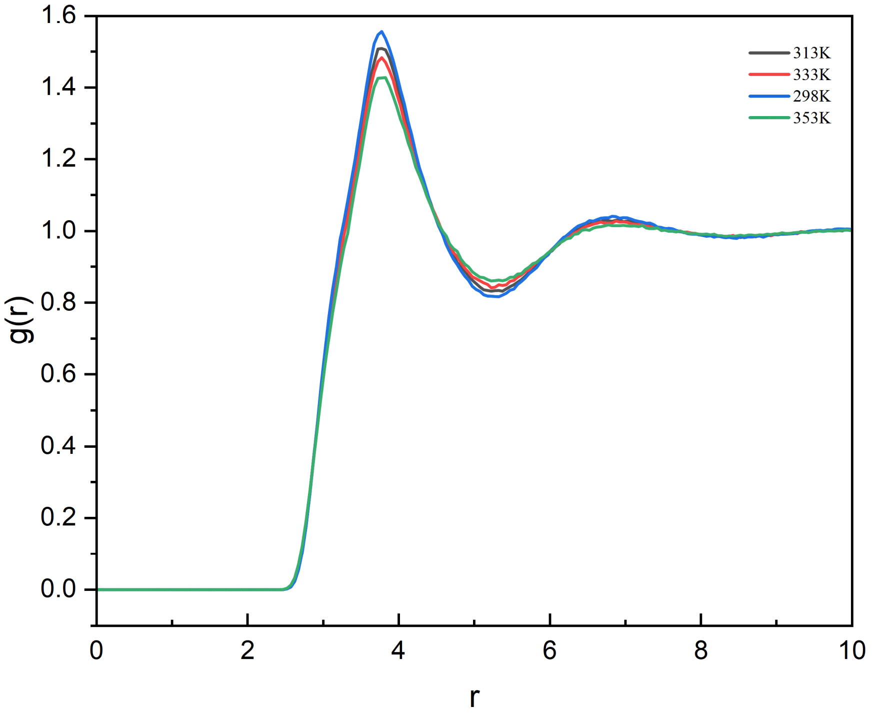

Figure S5. Radial distribution functions g(r) for CO_2–O(H_2O) at different temperatures (3.5 mol/L NaCl, 10 MPa).

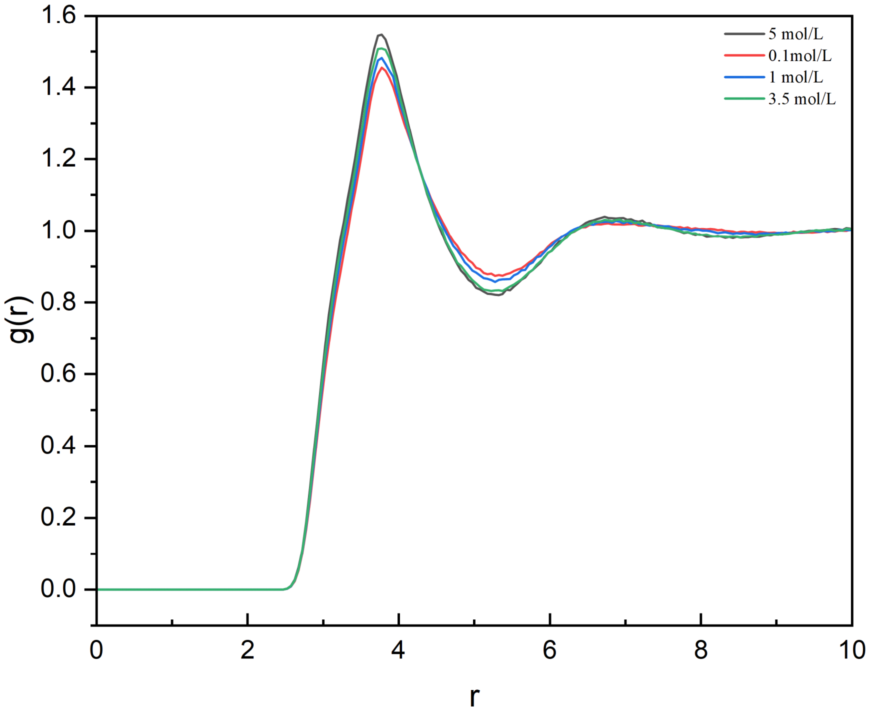

Figure S6. Radial distribution functions g(r) for CO_2–O(H_2O) at different NaCl concentrations (313 K, 10 MPa).

case	NaCl conc. (mol/L)	Temperature (K)	Pressure (MPa)	Diffusion coefficients (10^{-9} m²/s)	Block SD
1	3.5	313	10	0.37417	0.095
2	0.1	313	10	0.68278	0.050
3	1	313	10	0.41621	0.115
4	5	313	10	0.34369	0.071
5	3.5	298	10	0.26895	0.024
6	3.5	333	10	0.45065	0.027
7	3.5	353	10	0.67009	0.077
8	3.5	313	3	0.36016	0.089
9	3.5	313	30	0.38733	0.059
10	3.5	313	40	0.32906	0.079

Table S1. Detailed diffusion coefficients of CO_2 under all simulated conditions (salinity, temperature, pressure) with standard deviations.
